# Influence of water salinity on corrosion risk—the case of the southern Baltic Sea coast

**DOI:** 10.1007/s10661-014-3744-3

**Published:** 2014-04-03

**Authors:** K. Zakowski, M. Narozny, M. Szocinski, K. Darowicki

**Affiliations:** Department of Electrochemistry, Corrosion and Materials Engineering, Chemical Faculty, Gdansk University of Technology, 11/12 Narutowicza St, 80-233 Gdansk, Poland

**Keywords:** The Baltic Sea, Seawater, Corrosion rate, Water corrosivity, Salinity

## Abstract

Water corrosivity in Gdansk Bay, Poland, the southern part of the Baltic Sea, was investigated. The analysed region is heavily industrialized, and the coastline is very diverse. Twenty-seven test points along the coastline were selected. Water parameters such as salinity, total dissolved solids content, resistivity, conductivity, oxygenation, pH and corrosion rate were determined. The results of the investigation are presented. Water samples were collected, and structural steel specimens were exposed in the water for 2 months. The corrosion rate for each test point was determined and plotted on a map. The spatial distribution of water parameters was calculated using the ‘inverse distance to a power’ method and presented on the maps. Salinity did not exceed 0.7 %, and average corrosion rate equalled 0.0585 mm/year.

## Introduction

From a chemical standpoint, seawater is an aqueous solution of salts. On average, ocean water salinity equals approximately 3.5 % (Williams et al. 2010). Salinity is evaluated by determination of [Cl^−^] ion concentrations in water. Empirical dependence, which states that salinity equals 1.80655 × [Cl^−^], is used. In practice, conductivity measurements are performed. Conductivity is converted into salinity by employing empirical relationships. A variety of salts compose ocean water, and their typical quantity is reflected in artificial ocean water. The composition of artificial ocean water according to the ASTM D1141-98(2013) ‘Standard Practice for the Preparation of Substitute Ocean Water’ is presented in Table 1.

**Table 1:** Artificial ocean water composition according to the ASTM D1141-98(2013)

	Salt concentration [g/dm^3^]	Salt [%]
NaCl	24.53	0.681
MgCl_2_	5.2	0.144
Na_2_SO_4_	4.09	0.114
CaCl_2_	1.16	0.0322
KCl	0.695	0.0193
NaHCO_3_	0.201	0.00558
KBr	0.101	0.00280
H_3_BO_3_	0.027	0.000749
SrCl_2_	0.025	0.000694
NaF	0.003	0.0000833
Ba(NO_3_)_2_	0.0000994	0.00000276
Mn(NO_2_)_2_	0.000034	0.000000944
Cu(NO_3_)_2_	0.0000308	0.000000855
Zn(NO_3_)_2_	0.0000096	0.000000266
Pb(NO_3_)_2_	0.0000066	0.000000183
AgNO_3_	0.00000049	0.0000000136

Differences in local salinity might be a result of the phenomenon of water evaporation, which increases salinity levels (Da-Allada et al. 2013). A limited water exchange in a given sea area and an uncompensated sweet water inflow result in a decrease in salinity (Zhang and Yan 2012; Rodhe and Winsor 2002). In comparison to most oceanic waters, the Baltic Sea exhibits low evaporation rates. The Baltic Sea is a marginal sea, and water exchange with the Atlantic Ocean is only possible through the narrow Danish straits. For these reasons, the Baltic Sea is considered to be a moderately salinized sea—approximately 1 % (Feistel et al. 2010). The salinity of Gdansk Bay is even lower—approximately 0.7–0.8 %—due to a high sweet water inflow, mainly from the Vistula River mouth near the Isle of Sobieszewo (Rak and Wieczorek 2012).

Due to the global economic and merchandise trade expansion, there is a major infrastructure development in Polish coastal areas. New facilities such as ports, harbours, oil storage-handling bases, piers, bridges and other stationary offshore structures are being built. These facilities are susceptible to the corrosive influence of seawater (Melchers 2006; Hajeeh 2003). Their anticorrosion protection systems mostly consist of mutually supplementary coating (Hou Jian et al. 2013) and cathodic protection systems (Hartt 2012; Zakowski 2011). The better the coating barrier properties are, the lower is the cathodic current density required to guarantee complete protection. Protection technology should be adjusted to the local degree of corrosion risk caused by the seawater environment (Al-Malahy and Hodgkiess 2003; Goldberg 1988). A properly designed corrosion protection system should assure complete corrosion resistance.

The corrosion rate in seawater is a function of a large number of mutually dependent factors. Corrosivity of natural water increases proportionally with salinity. If salinity exceeds 3 %, water corrosivity decreases (Kirk and Pikul 1990). This phenomenon is caused by the fact that corrosion rates tend to increase when water conductivity increases. The higher the salinity is, the lower the oxygen solubility is (Weiss 1970). Thus, above 3 % salinity, the corrosion rate in seawater decreases. The pH of oceanic water ranges from 7.9 to 8.1 (Rerolle et al. 2012). Lower-pH regions are situated near river mouths, for example. Higher-pH regions are where water blooms and tide pools are (Kharchenko et al. 2011; Wong and Oatts 1984). Within the range of the pH observed in seawater, the corrosion rate is approximately equal. Gases dissolve in water and the most important ones from the corrosion risk standpoint are oxygen and carbon dioxide. The solubility of oxygen and carbon dioxide decreases with an increase in temperature and salinity. Carbon dioxide influences water pH—it makes water more acidic (Rerolle et al. 2012; Ayers 1991). Oxygen acts as a depolarizer in the cathodic half-cell, and it enhances corrosion risk. The amount of dissolved oxygen is influenced not only by temperature, atmospheric pressure and water salinity (Debelius et al. 2009) but also by biological activity. For instance, photosynthesis processes, which occur close to the water surface, increase oxygen concentrations (Ayers 1991; Rose et al. 2003). Calcareous deposits (Zakowski et al. 2013; Neville and Morizot 2002; Zamanzade et al. 2007), deposition of corrosion products (Hu Jiayuan et al. 2013; Jia-Yuan et al. 2012; Liu et al. 2013), water contamination (Melchers 2009; 2007) and fouling also affect corrosion rates (Khana et al. 2013).

The aims of this paper are to evaluate corrosion risk and to present the typical water parameters (oxygenation, resistivity, total dissolved solids (TDS), salinity) of Gdansk Bay coastal areas, the southern part of the Baltic Sea. This part of the Polish coastline is greatly diversified. There is a great sweet and contaminated water inflow from the Vistula River mouth in the proximity of the Isle of Sobieszewo. The investigated area is heavily industrialized. There are two large shipyards in Gdynia and Gdansk, many smaller maritime companies, LOTOS S.A. oil refinery (Poland’s second greatest petrochemical plant), Siarkopol Gdansk (sulphur exporter), Gdanskie Zakłady Nawozow Fosforowych Fosfory Sp. z o.o. (phosphoric fertilizer producer), a Polish Navy harbour and shipyard and Gdynia and Gdansk Port (Altayaran and Madany 1992). The corrosivity of water heavily influences the integrity of these facilities.

## Material and methods

In selected places at the Gdansk Bay coastline, field measurements of typical parameters of water were performed. Water samples were collected in order to perform laboratory exposure of corrosion coupons and determine their corrosion rates. The coupons were made of S235JR structural steel. The composition of the S235JR steel is C 0.2 max, Mn 1.4 max, P 0.04 max, S 0.04 max, N 0.012 max and Cu 0.55 max.

### Field measurement locations

**Fig. 1:**
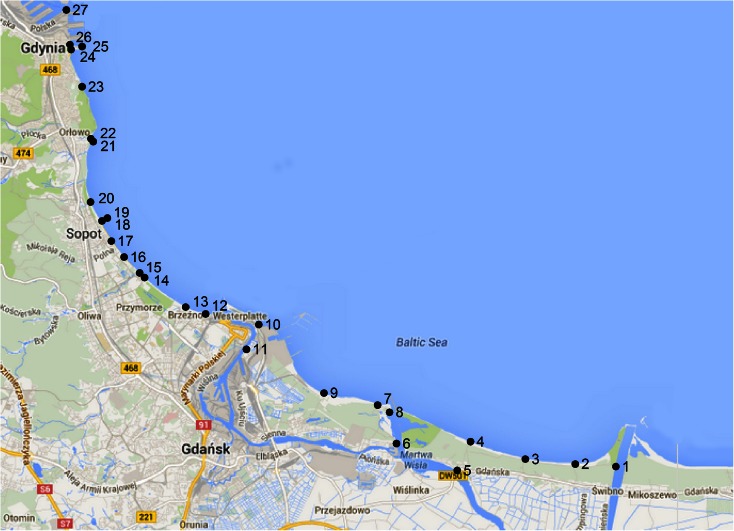
Test points along the Gdansk Bay coastline

Twenty-seven measurement points were chosen. They were located along the Gdansk Bay coastline, from the Vistula River mouth near the Isle of Sobieszewo (Gdansk) to the Port of Gdynia. The locations where the measurements and the water samples were taken are presented in Fig. 1. The description, localization and water parameters for each and every point are presented in Table 2.

**Table 2:** Set of values of measured parameters along the Gdansk Bay coastline

Sample number	Location	Longitude	Latitude	Temperature [C]	Oxygenation [g/mg/dm^3^]	Conductivity [μS/cm]	Resistivity [Ω cm]	TDS [ppm]	Salinity [‰]	Corrosion rate [mm/year]	pH
1	The Vistula River dug-through	18.939694	54.345051	13.04	6.74	474	2,108.71	237	0.230	0.1061	8.36
2	The Isle of Sobieszewo	18.910769	54.346101	7.50	9.96	9,668	103.33	4,834	5.442	0.0578	8.45
3	The Isle of Sobieszewo	18.875186	54.348152	7.77	8.63	9,746	102.67	4,873	5.493	0.0827	8.33
4	The Isle of Sobieszewo	18.83601	54.355381	7.38	8.23	10,207	98.17	5,103	5.768	0.0827	8.46
5	The Dead Vistula River—pontoon bridge	18.826569	54.34345	10.28	9.31	7,569	132.17	3,785	4.203	0.0802	8.94
6	The Bold Vistula River	18.783295	54.354562	9.25	10.15	8,698	115.00	4,349	4.872	0.0804	8.97
7	Gorki Zachodnie	18.769835	54.370509	7.54	9.76	9,740	103.00	4,870	5.487	0.0834	8.66
8	Gorki Zachodnie—marina	18.778285	54.367571	8.48	9.17	8,564	117.00	4,282	4.790	0.0398	8.65
9	Stogi	18.731579	54.375521	7.91	8.17	10,423	95.83	5,211	5.910	0.0404	8.45
10	Westerplatte	18.685016	54.403882	7.65	8.24	11,177	89.33	5,588	6.360	0.0403	8.55
11	Martwa Wisła	18.67624	54.393652	8.99	8.42	7,604	131.50	3,802	4.220	0.0376	7.88
12	Nowy Port	18.64714	54.408266	7.77	8.99	11,280	89.00	5,640	6.430	0.0419	8.63
13	Brzezno	18.632897	54.411132	8.32	8.68	11,213	89.00	5,607	6.390	0.0415	8.50
14	Jelitkowo	18.603671	54.423337	10.15	9.43	11,188	89.25	5,594	6.390	0.0511	8.83
15	Jelitkowo	18.600099	54.425278	8.28	9.43	9,574	1.50	4,787	5.392	0.0393	8.65
16	Sopot Hestia	18.589022	54.431869	8.02	10.13	11,242	89.00	5,620	6.406	0.0517	8.80
17	Sopot	18.579959	54.438477	8.05	10.67	11,452	87.00	5,726	6.534	0.0510	8.80
18	Sopot—pier	18.573224	54.446837	5.47	9.70	11,762	85.20	5,852	6.652	0.0546	8.71
19	Sopot—marina	18.577108	54.448051	6.60	10.24	11,318	88.20	5,659	6.436	0.0540	8.96
20	Sopot Kamienny Potok	18.56511	54.454697	7.81	10.11	11,524	87.20	5,763	5.574	0.0551	8.87
21	Gdynia Orłowo	18.565274	54.480914	6.73	10.96	11,480	87.00	5,740	6.540	0.0590	9.78
22	Gdynia Orłowo—pier	18.566947	54.479719	6.64	10.27	11,512	87.00	5,756	6.550	0.0610	8.94
23	Gdynia Coastwalk	18.559039	54.502433	6.65	9.99	11,698	85.00	5,850	6.670	0.0590	8.85
24	Beniowski Quay	18.551197	54.517868	7.98	9.91	11,456	87.00	5,727	6.530	0.0540	8.66
25	President Harbour Basin	18.559007	54.519107	6.76	10.83	11,532	87.00	5,766	6.570	0.0550	8.99
26	Pomorski Harbour Basin	18.550381	54.519911	5.86	10.48	11,688	86.00	5,844	6.650	0.0560	8.85
27	French Quay	18.547837	54.534243	4.68	9.57	11,940	84.00	5,970	6.780	0.0630	8.65

### Investigated water parameters

At every test point (Fig. 1), the following seawater parameters were measured:

- Temperature [°C]
- Atmospheric pressure [hPa]
- pH
- Dissolved oxygen content [mg/dm^3^]
- Water resistivity [Ω cm]/water conductivity [μS/cm]
- TDS content [ppm]
- Salinity [‰]

As the temperature decreases, the maximum salt solubility also decreases (Helber et al. 2012; Bingham et al. 2010), which causes seawater to exhibit lower conductivity and higher electrolyte resistivity (Sharqawy 2013; Sasidhar and Vijay Kumar 2008). The lower the temperature is, the greater the oxygen solubility in water is and the greater the corrosion rate may be.

Atmospheric pressure has almost no influence on atmospheric corrosion rate due to its limited variation range. In the case of immersed structures, the pressure varies depending on the depth, and the pressure factor is more significant—it might cause corrosion cracking. pH (activity of H_3_O^+^ ions) is important when a corrosion protection system is designed due to its effect on the thermodynamic stability of water.

The corrosion rate in seawater is controlled by an oxygen reduction reaction. The oxygen inflow to the cathode is a function of oxygen concentrations and water flow. Thus, higher corrosion rates are observed in splash zones and in the locations where waving is greater due to increased oxygenation and enhanced oxygen migration to the structure’s surface (Al-Fozan and Malik 2008).

Electrolyte conductivity increases with the temperature due to enhanced ion mobility. It can be assumed that the lower the electrolyte electric resistance is, the lower the corrosion rate is. The type of corrosion attack also depends on resistivity. For instance, in low-electric-resistivity electrolytes, the galvanic corrosion process can take place at a greater distance from the metal-metal contact, as compared to the electrolytes of higher electric resistivity.

The TDS parameter quantifies both organic and inorganic compounds in the ionic, particle or colloidal states (Boerlage 2012). Water can be regarded as fresh (TDS < 1,500 ppm), brackish (1,500 < TDS < 5,000) or saline (TDS > 5,000).

Salinity mainly influences water resistivity. Furthermore, dissolved salts, especially Cl^−^, are unfavourable for passive metals and may cause local passive layer failures initiating pitting corrosion (Ylasaari et al. 1997).

Performed tests do not take variable hydrodynamic conditions such as storms and swells into account. They increase the instantaneous corrosion rate, but their effect on yearly averaged corrosion rate is not very significant. Sprouting and microbial activity were not investigated.

Field measurements presented in this article were carried out in May 2013 and lasted 2 days. A Hanna HI 9828 meter was used. It consisted of three probes. In order to investigate the concentration of dissolved oxygen, a galvanic Ag/Zn probe was used. The TDS content, salinity and electric conductivity/resistivity were measured with the conductivity meter, the electrodes of which were made of AISI 316 steel. At each and every measurement point, the probes were immersed 0.5 m deep, approximately 5 m away from the coastline. Data logging was executed after 5 min of probe immersion. Ten measurements were taken for 5 min at 30-s intervals.

### Corrosion rate determination

Gravimetric measurements were performed in order to determine corrosion rates of steel coupons immersed in water sampled at every test point. Coupons 100 × 50 mm were made of a S235JR 2-mm-thick carbon steel sheet. The coupons were grounded to the Sa1 steel surface preparation grade. The samples were exposed in individual containers to unstirred water at room temperature (three coupons for every test point). The exposure lasted 2 months. Then, the samples were washed under running water. Corrosion products were removed in 10 % hydrochloric acid mixed with 9 g/dm^3^ hexamethylenetetramine (CH_2_)_6_ N_4_ corrosion inhibitor. The samples were washed, dried and weighted. Mass loss measurements were performed with 0.0001-g accuracy. For calculations, an averaged mass loss of the three exposed coupons was used.

Corrosion rate was determined using the following relationship: ${v}_{\mathrm{corr}}=\frac{\varDelta \overline{m}}{S\cdot t},$

where Δ$\overline{m}$ is the averaged coupon weight loss [g], *S* is the total coupon surface area [cm^2^] and *t* is the exposure time [years]. *V*_corr_ was divided by sample density in order to obtain the linear corrosion rate expressed in the generally accepted unit [mm/year].

## Results and discussion

A set of parameters—longitude, latitude, temperature, oxygenation, conductivity, resistivity, TDS, salinity, corrosion rate and pH—are presented in Table 2. Presented values are the average of ten measurements taken at 30-s intervals. The test points described by their local name (Table 2, location) and their exact locations are given in the North American Datum 1983 (NAD83) system (Table 2; longitude, latitude). The maps have been proposed, and isolines representing water parameters have been calculated using the ‘inverse distance to a power’ method. The ‘inverse distance to a power’ method is slightly different from the ‘inverse distance’ method. Instead of a distance between the measurement point and the grid node, a power of that distance is calculated. The greater the power coefficient is, the greater the significance of the points closer to the calculated grid node is.

**Fig. 2:**
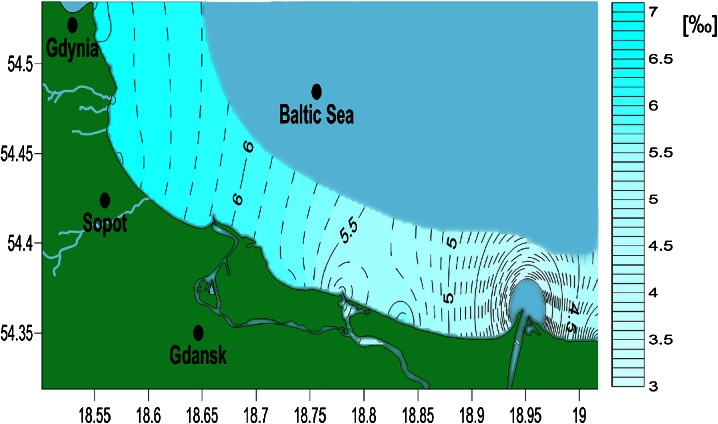
Map of salinity distribution along the Gdansk Bay coastline

The distribution of water salinity in the investigated region is presented in Fig. 2. On the OX and OY axes, the longitude and the latitude are presented in the NAD83 system, respectively. The further away from the Vistula River mouth one moves, the higher the salinity is. The Vistula River is a sweet water source for the Isle of Sobieszewo area. Local salinity is also influenced by smaller watercourses and rivers, for instance in Jelitkowo (Table 2, sample no. 15). In the areas located further away from watercourses, the salinity is approximately the same, for instance in Gdynia (Table 2, sample nos. 21–27). The Vistula River dug-through is going to be greatly developed in upcoming years as a part of the Polish Ministry of Infrastructure programme ‘Program rozwoju infrastruktury transportu wodnego srodladowego w Polsce’—‘Program of inland water transport infrastructure development in Poland’.

**Fig. 3:**
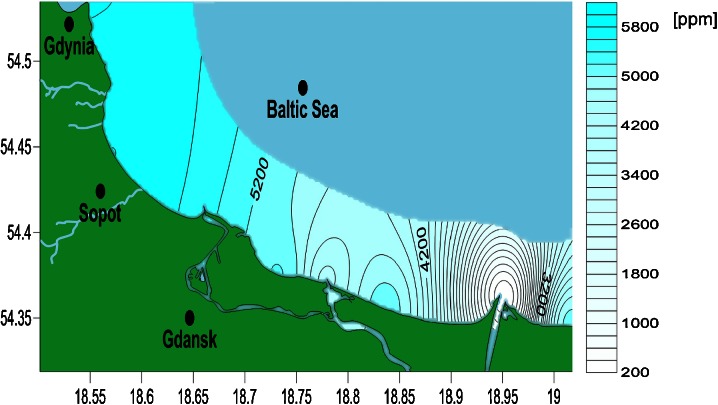
Map of TDS distribution along the Gdansk Bay coastline

**Fig. 4:**
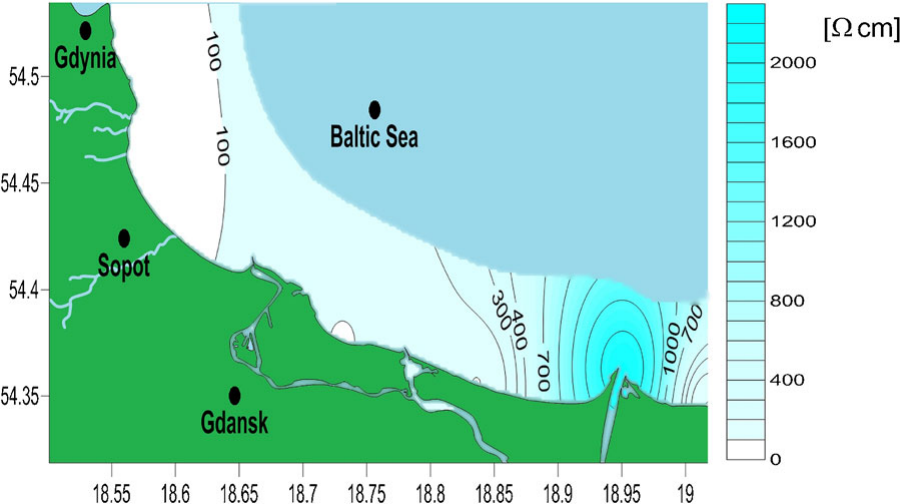
Map of resistivity distribution along the Gdansk Bay coastline

The distribution of TDS along the Gdansk Bay coastline is presented in Fig. 3, and resistivity is presented in Fig. 4. The TDS content is well correlated with resistivity (Pearson correlation coefficient = −0.85). The reason for such a strong correlation is the use of the same probe for both measurements.

**Fig. 5:**
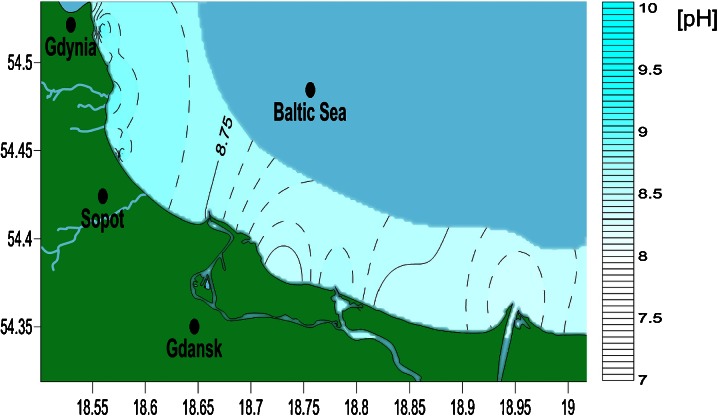
Map of seawater pH distribution along the Gdansk Bay coastline

The pH of seawater in Gdansk Bay is presented in Fig. 5. Only at two test points was water pH out of the 8–9 range. In the Dead Vistula River (Table 2, sample no. 11), the water was slightly more acidic and the pH was equal to 7.88. In Gdynia Orlowo (test point 21), the pH was slightly higher and was equal to 9.78. The measurement was taken inside the bay, where the water is rather unstirred and there is a small polluted river nearby, which might enhance biological activity leading to an increase in the pH of the water.

**Fig. 6:**
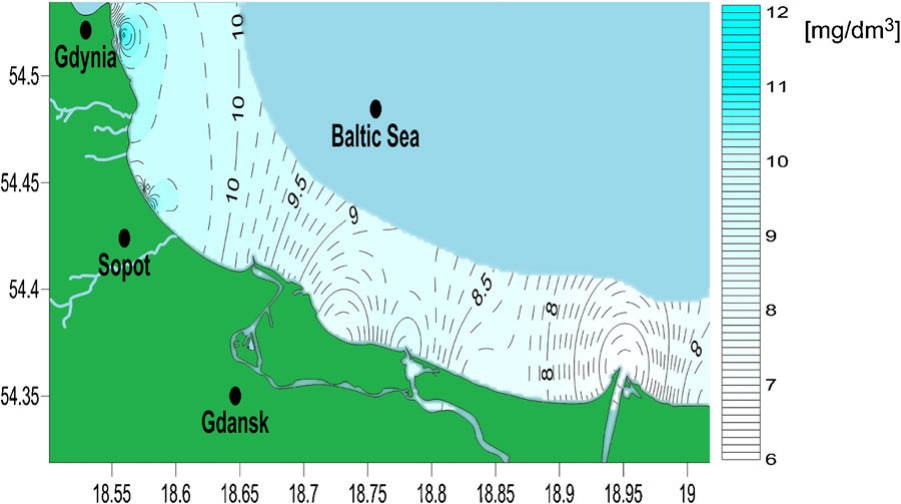
Map of seawater oxygenation distribution along the Gdansk Bay coastline

The oxygenation of seawater in the investigated area is presented in Fig. 6. The lowest oxygenation value was equal to 6.74 mg/dm^3^ and was observed at the Vistula River mouth. The Vistula River is notoriously reported to be polluted at its river mouth. Bacterial pollution from municipal sewage, chemical plant sewage and fertilizer run-off might be the causes of the very low water oxygenation (Environment and United Nations Environment Programme Global 2007).

**Fig. 7:**
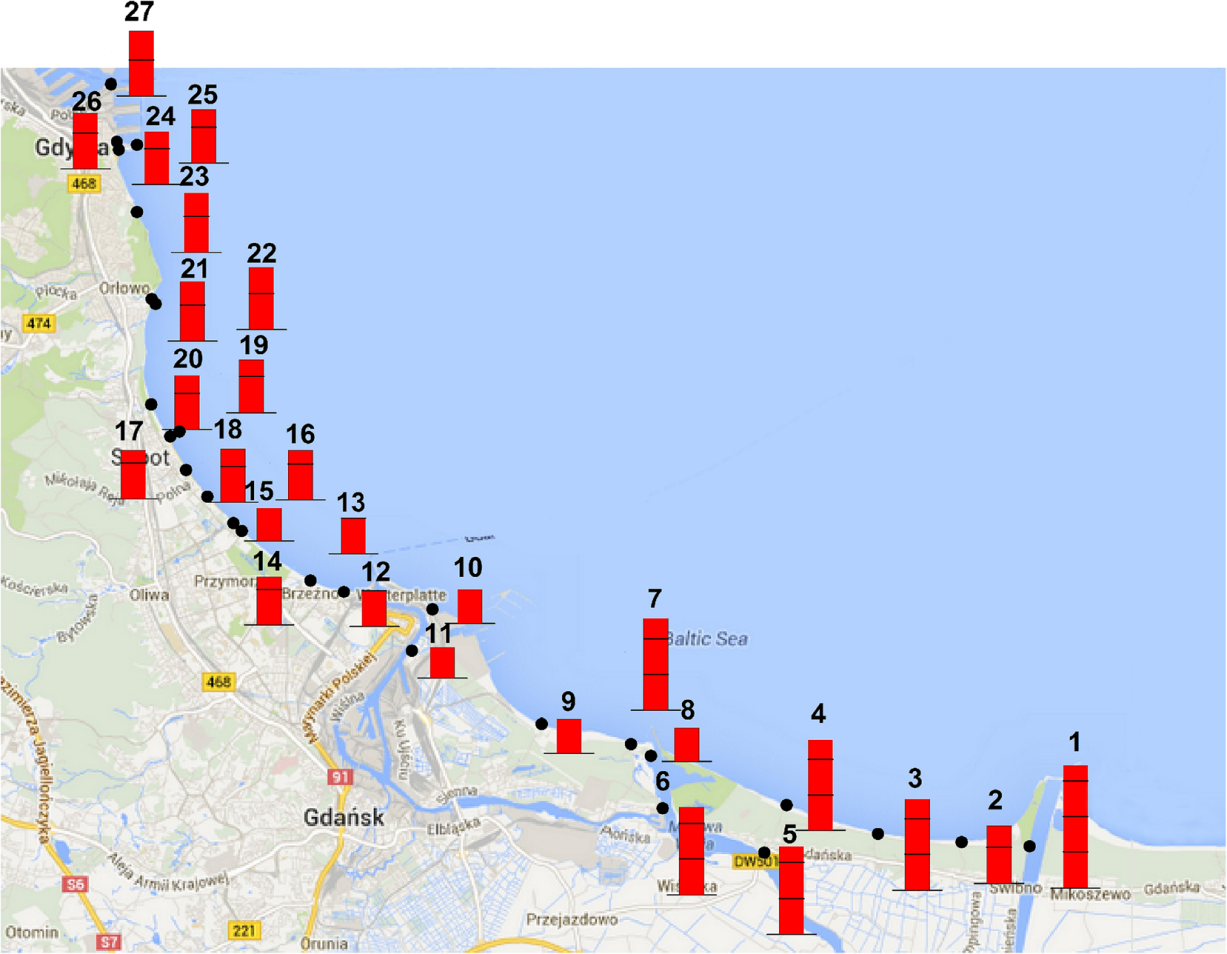
Map of relative corrosion rates along the Gdansk Bay coastline

The determined corrosion rates for all test points ranged from 0.035 to 0.1061 mm/year, and their values are presented in Fig. 7. The obtained corrosion rates are lower than typical corrosion rates for oxygenated 3.5 % seawater, which were equal to approximately 0.5 mm/year and in extreme cases 1.0 mm/year (Gardiner and Melchers 2003). Thus, the low salinity of seawater has a positive effect from the standpoint of corrosion risk of offshore structures. It has to be noted that locally where the water flows, string and oxygenation are high and the corrosion rate might rise significantly.

The highest corrosion rate among investigated samples was recorded for the Vistula River dug-through (Table 2, sample no. 1) and was equal to 1.061 mm/year. Yet, water resistivity, obviously due to the proximity of the river mouth, was the highest, and the salinity was the lowest. If only those parameters were concerned, one would expect the opposite effect—lower corrosion rates as compared to the other test points. Thus, the other factors must have had an effect on corrosion aggressiveness in that area. One can assume that it was a water pollution factor, which was not investigated in this study. The further away from the Vistula River mouth one goes, the lower the corrosion rates are, probably due to the mixing of seawater with sweet water from the river. The corrosion rate determined for water sampled from the Dead Vistula River (Table 2, sample no. 11) was the least corrosive. Apart from the Vistula River dug-through, it also had the lowest resistivity.

In general, the corrosion rates obtained for the Gdansk area are more varied than the rates for the Gdynia/Sopot area. The variation of results might be caused by the presence of the Vistula River arms and mouth. The corrosion rates determined for the Gdynia and Sopot areas vary between 0.05 and 0.06 mm/year. A low discrepancy of the measurements might occur due to the presence of fewer and smaller watercourses.

The corrosion rates determined for the Gdansk Bay coastal area are in accordance with Wall and Wadsö results for the steel sheet pile walls in the harbour of Halmstad on the Swedish west coast where average corrosion rates varied between 0.02 and 0.06 mm/year (Wall and Wadsö 2013). Wall and Wadsö determined corrosion rates by steel sheet pile thickness measurements after decades of immersion in seawater and referring the results to the initial steel sheet pile thickness.

The results indicate that steel offshore structures located in the southern Baltic area require anticorrosion protection systems. This fact has to be taken into account when a new infrastructure is designed. For instance, according to EUROCODE 3, the corrosion rate of 0.08 mm/year corresponds to ‘seawater in the zone of high attack’ and 0.05 mm/year or lower to ‘polluted sweet water in the zone of high attack’ and ‘seawater in the zone of permanent immersion’.

Another issue is the current condition of already operating offshore infrastructures. Quays are designed to have a life cycle of 50 to 100 years. There was a rapid offshore infrastructure development in Poland between the years 1950 and 1970. Thus, periodical control of the infrastructure condition is recommended.

## Conclusions

The following conclusions can be drawn from the obtained results:

- The results obtained for the southern Baltic coastal area indicate that in low-salinity seas, as compared to oceans, the corrosion risk is significantly lower. The average salinity in the coastline of Gdansk Bay did not exceed 0.7 %, and the average steel corrosion rate was equal to 0.0585 mm/year. In the proximity of the Vistula River mouth, the corrosion aggressiveness of water increases and the corrosion rate of structural steel was equal to approximately 0.08 mm/year in Gdansk Bay and approximately 0.1 at the river mouth.
- Corrosion aggressiveness of seawater not only is a function of water resistivity and salinity, but it also is influenced by other mutually supplementary factors such as temperature, pH reaction, oxygenation, water flow, dissolved gas content and pollutants (McNeill and Edwards 2002). In Gdansk Bay, those factors are connected with river pollution, the distance from sweet water sources and industrial infrastructure.
- Offshore structures require corrosion protection systems. Adequate precautions should be taken when the construction is being designed. An anticorrosion system should involve proper selection of materials as well as application of adequate paint systems aided by cathodic protection.
